# A cross-sectional study of latent tuberculosis infection, insurance coverage, and usual sources of health care among non-US-born persons in the United States

**DOI:** 10.1097/MD.0000000000024838

**Published:** 2021-02-19

**Authors:** Esther Annan, Erica L. Stockbridge, Dolly Katz, Eun-Young Mun, Thaddeus L. Miller

**Affiliations:** a Department of Biostatistics and Epidemiology, School of Public Health, University of North Texas Health Science Center, TX; b Department of Advanced Health Analytics & Solutions, Magellan Healthcare, Magellan Health, Inc., Scottsdale, AZ; c Division of Tuberculosis Elimination, National Center for HIV/AIDs, Viral Hepatitis, STD, and TB Prevention, US Centers for Disease Control and Prevention, GA; d Department of Health Behavior and Health Systems, School of Public Health, University of North Texas Health Science Center, TX.

**Keywords:** health care delivery, health care systems, health insurance, latent tuberculosis infection, preventive health services, tuberculosis

## Abstract

More than 70% of tuberculosis (TB) cases diagnosed in the United States (US) occur in non-US-born persons, and this population has experienced less than half the recent incidence rate declines of US-born persons (1.5% vs 4.2%, respectively). The great majority of TB cases in non-US-born persons are attributable to reactivation of latent tuberculosis infection (LTBI). Strategies to expand LTBI-focused TB prevention may depend on LTBI positive non-US-born persons’ access to, and ability to pay for, health care.

To examine patterns of health insurance coverage and usual sources of health care among non-US-born persons with LTBI, and to estimate LTBI prevalence by insurance status and usual sources of health care.

Self-reported health insurance and usual sources of care for non-US-born persons were analyzed in combination with markers for LTBI using 2011–2012 National Health and Nutrition Examination Survey (NHANES) data for 1793 sampled persons. A positive result on an interferon gamma release assay (IGRA), a blood test which measures immunological reactivity to *Mycobacterium tuberculosis* infection, was used as a proxy for LTBI. We calculated demographic category percentages by IGRA status, IGRA percentages by demographic category, and 95% confidence intervals for each percentage.

Overall, 15.9% [95% confidence interval (CI) = 13.5, 18.7] of non-US-born persons were IGRA-positive. Of IGRA-positive non-US-born persons, 63.0% (95% CI = 55.4, 69.9) had insurance and 74.1% (95% CI = 69.2, 78.5) had a usual source of care. IGRA positivity was highest in persons with Medicare (29.1%; 95% CI: 20.9, 38.9).

Our results suggest that targeted LTBI testing and treatment within the US private healthcare sector could reach a large majority of non-US-born individuals with LTBI. With non-US-born Medicare beneficiaries’ high prevalence of LTBI and the high proportion of LTBI-positive non-US-born persons with private insurance, future TB prevention initiatives focused on these payer types are warranted.

## 1 Introduction

Preventing tuberculosis (TB) in the non-US-born United States (US) population is a national public health priority. More than 70% of TB cases diagnosed in the US occur in non-US-born persons; the same population has experienced less than half the recent TB incidence rate declines of US-born persons (declining 1.5% vs 4.2%, respectively, from 2018 to 2019).^[1]^ Approximately 90% of TB cases in non-US-born persons arise not from recent transmission but from reactivation of latent TB infection (LTBI), most likely acquired in their countries of origin.^[2]^ Accordingly, identification and treatment of LTBI in persons from high TB incidence countries is critical to TB prevention and elimination and is recommended by clinical practice guidelines.^[3,4]^

In the US, LTBI-related services are most often delivered in local public health departments but such organizations lack the capacity to provide the volume of services needed to implement current recommendations.^[5,6]^ Conversely, private sector providers in the US (e.g., private physicians, community health centers) may have the capacity to initiate targeted LTBI testing and treatment. However, the success of initiatives to increase these TB prevention activities within the private health care sector depends on LTBI-positive non-US-born persons’ access to health insurance and health care providers. While most non-US-born persons have health insurance and/or a usual source of health care,^[7,8]^ the health care access of non-US-born persons with LTBI is unknown. We conducted descriptive analyses of data from the 2011 to 2012 National Health and Nutrition Examination Survey (NHANES) to assess TB prevention opportunities in the private health care sector.

## 2 Methods

### 2.1 Data source and study design

NHANES is a biennial cross-sectional survey of a nationally representative sample of civilian noninstitutionalized individuals in the US. This survey is conducted by the National Center for Health Statistics of the US Centers for Disease Control and Prevention. It uses a complex, multistage probability sampling design; data are collected from interviews and physical examinations.^[9]^ The 2011–2012 NHANES dataset, which is the most recent survey with TB-related questions and laboratory measurements, was used for this analysis. This project was reviewed and approved by the North Texas Regional Institutional Review Board as exempt category research.

Our study included non-US-born noninstitutionalized respondents ages 6 years or older who had interferon gamma release assay (IGRA) test results and non-missing data for self-reported insurance and usual source of healthcare variables. IGRAs are whole-blood tests that are used to diagnose LTBI by evaluating cell-mediated immune response to *Mycobacterium tuberculosis*.^[10]^ The 2011–2012 NHANES tested for LTBI in persons aged ≥6 years with a tuberculin skin test and a QuantiFERON-TB Gold In-Tube IGRA.^[11]^ We used IGRA results because persons with prior Bacille Calmette–Guerin (BCG) vaccines may experience false positive results with tuberculin skin tests , and IGRAs more accurately predict the progression of LTBI to TB in non-US-born persons.^[12]^ Those below age 6 years were excluded because IGRAs are not recommended for young children.^[13]^ IGRA results were available for 91.7% of non-US-born respondents aged ≥6 years who received physical examinations.

### 2.2 Study variables

IGRA results were categorized as positive or negative; IGRA positivity was used as a proxy for infection. In addition to IGRA positivity, our study focused on health insurance and usual sources of health care. We created a health insurance variable that categorized coverage as Medicare, Medicaid/Children's Health Insurance program, private insurance, other or unspecified insurance, or no insurance (Supplemental File 1). Our categorical usual source of health care variable included no usual source of care, clinic/health center, doctor's office/health maintenance organization (HMO), and other/not specified. The “no usual source of care” category included persons who said they had no usual source of care and those answering, “hospital emergency room.” “Other/not specified” included respondents giving their usual source of care as “hospital outpatient department,” “some other place,” and those with an unspecified usual source of care (Supplemental File 1).

Demographic variables that were evaluated in this study included gender and age. Participants were identified as either male or female and age was categorized as 6 to 34 years, 35 to 49 years, 50 to 64 years, and 65 years or older. Duration of US residence for non-US-born individuals was categorized as being present in the US for less than 5 years, 5 years or more, or unspecified; this categorization reflects increasing probability for having insurance, a key driver of healthcare utilization.^[14]^ Federal poverty level (FPL) was categorized as above 137%, less than or equal to 137%, and missing to reflect Medicaid eligibility thresholds in expansion states.^[15]^

### 2.3 Statistical analysis

Univariate analysis was performed to evaluate the demographic characteristics of the sample. These variables were further examined for LTBI prevalence by each subcategory in a bivariate analysis. We estimated demographic category percentages by IGRA status, IGRA percentages by demographic category, and 95% confidence intervals for each percentage; these estimates yielded the sample distribution and IGRA positivity by category. The relative standard error (RSE) for each estimate was calculated and the number of observations was evaluated to assess the reliability of each estimate. In accordance with NHANES analytic guidelines, estimates for which the RSE exceeded 30% and/or the number of observations was less than 30 were deemed unreliable and notated as such in tables.^[9,16]^

We used Stata SE 15.1 (StataCorp) to adjust for weights and complex survey design (Supplemental File 2).

## 3 Results

### 3.1 Demographic characteristics

Data for 1793 respondents were analyzed using NHANES weights to represent the total population of non-US-born persons aged ≥6 years. After weighting, an estimated 15.9% [95% confidence interval (CI) = 13.5, 18.7] of non-US-born persons were IGRA-positive. Respondents aged greater than or equal to 65 years were most often IGRA positive (32.0%, 95% CI: 23.6, 41.7%) (Table 1). Males and those with FPL less than or equal to 137 also had disproportionately higher IGRA positivity within the sample at 17.5% (95% CI: 14.5, 20.5) and 16.6% (95% CI: 12.3, 21.2), respectively (Table 1).

**Table 1:** Weighted estimates of IGRA positivity by demographic and health system characteristics of Non-US born persons in the US aged > = 6 years in 2011–2012.

Sociodemographic and health system characteristics	IGRA positive prevalence, % (95% CI) [unweighted # in numerator]
Total	15.9 (13.5, 18.7) [345]
Age	
6–34 years	7.6 (4.7, 11.9) [43]
35–49 years	14.6 (11.3, 18.6) [82]
50–64 years	25.8 (19.9, 32.6) [134]
> = 65 years	32.0 (23.6, 41.7) [86]
Gender	
Male	17.5 (14.5, 20.8) [192]
Female	14.3 (11.6, 17.5) [153]
Duration of Time in the US	
Lived in the US less than 5 years	10.2 (6.4, 15.9) [34]
Lived in the US 5 years or more	16.7 (13.9, 19.9) [297]
Federal Poverty Level	
≤ 137%	16.3 (12.3, 21.2) [142]
> 137%	14.8 (12.4, 17.5) [143]
Usual Source of Health Care Category	
None^1^	15.3 (11.9, 19.4) [81]
Any	16.1 (13.6, 19.0) [264]
Clinic/Health Center	17.3 (12.7, 23.0) [93]
Dr. Office/HMO	15.9 (13.3, 18.9) [165]
Other/Not Specified ^2^	7.0 (2.3, 19.1)^∗^^,^^†^ [6]
Health Insurance	
None	15.3 (11.8, 19.7) [116]
Any	16.2 (13.6, 19.3) [229]
Private Insurance	13.7 (10.8, 17.4) [112]
Medicare	29.1 (20.9, 38.9) [57]
Medicaid/CHIP	12.7 (7.2, 21.5) ^†^ [29]
Other/Unspecified	19.5 (11.5, 31.1) [31]
Combination of Health Insurance and Usual Source of Health Care (USHC) Category	
Neither Health Insurance Nor USHC	15.4 (11.1, 20.8) [59]
Either Health Insurance and/or USHC	16.0 (13.7, 18.7) [286]
Both Insurance and USHC	16.4 (13.5, 19.8) [207]
No Insurance but USHC	15.3 (10.9, 21.1) [57]
No USHC but Insurance	15.1 (9.6, 23.0) ^†^ [22]

### 3.2 Health insurance and usual source of health care

Most IGRA-positive non-US-born persons had private health care access as measured by having insurance or a usual source of health care (Table 2). Specifically, 74.1% (95% CI = 69.2, 78.5), 63.0% (95% CI = 55.4, 69.9), and 55.7% (95% CI = 48.5, 58.9) had a usual source of care, health insurance, or both, respectively. Relatively few, 18.7% (95% CI = 15.1, 22.8), had neither. Of the IGRA-positive persons, the most frequently noted usual source of care was “Dr. Office/HMO” (44.6%; 95% CI: 37.2, 52.2). Healthcare access patterns for IGRA negative non-US-born persons were similar to those with positive results. IGRA positivity was highest in persons with Medicare (29.1%; 95% CI: 20.9, 38.9) (Table 1).

**Table 2:** Weighted estimates of demographic and health system characteristics of Non-US-born persons in the US aged > = 6 years in 2011–2012, by interferon gamma release assay (IGRA) test results.

	Distribution by IGRA results (Column %)	
Sociodemographic and health system characteristics	IGRA-positive persons, % (95% CI) [unweighted # in numerator]	IGRA-negative persons, % (95% CI) [unweighted # in numerator]
Total	100 [345]	100 [1448]
Age		
6–34 years	18.3 (12.1, 26.9) [43]	38.4 (33.6, 43.5) [579]
35–49 years	29.0 (23.1, 35.6) [82]	31.5 (28.0, 35.2) [377]
50–64 years	33.0 (28.0, 38.4) [134]	20.4 (16.9, 24.3) [322]
> = 65 years	19.7 (14.4, 26.3) [86]	9.8 (8.0, 11.9) [170]
Gender		
Male	54.4 (49.7 58.9) [192]	49.4 (46.6, 52.1) [706]
Female	45.6 (41.1, 50.3) [153]	50.6 (47.9, 53.4) [742]
Duration of Time in the US		
Lived in the US less than 5 years	9.2 (6.3, 13.4) [34]	15.3 (11.4, 20.3) [232]
Lived in the US 5 years or more	86.2 (80.3, 90.5) [297]	81.2 (75.8, 85.6) [1172]
Federal Poverty Level		
≤ 137%	42.1 (31.9, 53.0) [142]	40.9 (34.4, 47.6) [656]
> 137%	42.1 (35.6, 48.9) [143]	45.9 (40.2, 51.7) [578]
Usual Source of Health Care Category		
None ^1^	25.9 (21.5, 30.8) [81]	26.9 (23.9, 30.1) [358]
Any	74.1 (69.2, 78.5) [264]	73.1 (69.9, 76.1) [1090]
Clinic/Health Center	28.5 (21.4, 36.8) [93]	26.2 (21.8, 31.1) [373]
Dr. Office/HMO	44.6 (37.2, 52.2) [165]	44.6 (38.4, 51.0) [678]
Other/Not Specified ^2^	1.0 (0.3, 3.0) ^∗^^,^^†^ [6]	2.3 (1.5, 3.5) [39]
Health Insurance		
None	37.0 (30.1, 44.6) [116]	38.3 (33.0, 44.1) [501]
Any	63.0 (55.4, 69.9) [229]	61.6 (55.9, 67.0) [947]
Private Insurance	33.1 (26.9, 40.0) [112]	38.3 (32.9, 43.9) [576]
Medicare	14.3 (10.4, 19.5) [57]	7.9 (6.4, 9.8) [131]
Medicaid/CHIP	6.5 (3.4, 12.2) ^∗^^,^^†^ [29]	8.4 (5.3, 13.1) [136]
Other/Unspecified	9.0 (5.5, 14.6) [31]	7.3 (5.2, 10.2) [104]
Combination of Health Insurance and Usual Source of Health Care (USHC) Category		
Neither Health Insurance Nor USHC	18.7 (15.1, 22.8) [59]	19.3 (16.2, 22.8) [237]
Either Health Insurance And/or USHC	81.3 (77.2, 84.9) [286]	80.7 (77.2, 83.8) [1211]
Both Insurance and USHC	55.7 (48.5, 58.9) [207]	54.0 (49.2, 58.8) [826]
No Insurance but USHC	18.4 (13.5, 24.5) [57]	19.1 (15.9, 22.7) [264]
No USHC but Insurance	7.2 (4.0, 12.7) ^†^ [22]	7.6 (5.6, 10.1) [121]

## 4 Discussion

These results have implications for both public health practice and private sector medical care. More than 70% of TB cases in the US occur in non-US-born persons^[1]^ and roughly 90% of these cases are attributable to reactivation of remotely-acquired LTBI.^[2]^ These statistics, along with our findings, suggest a need for public health agencies to engage with private sector health care providers and payers who serve non-US-born persons. Arrangements in which public health practitioners or consulting medical experts provide technical support to private sector providers to increase targeted testing and treatment could accelerate domestic TB elimination efforts. Our finding that nearly two-thirds of non-US-born individuals with LTBI have health insurance suggests that payers can facilitate LTBI care, with financial benefit to providers.

Policy change can also expand TB prevention opportunities in the private sector. Because the US Preventive Services Task Force has given LTBI testing of high-risk persons a “B” rating,^[4]^ the Affordable Care Act (ACA) mandates that such testing be covered by most private health insurance plans with no patient cost sharing. Similarly, the Centers for Medicaid and Medicare Services (CMS), which finances care for over 100 million people, has the option to cover testing without cost sharing. Lower out-of-pocket costs increase the use of preventive care,^[17]^ so these policies could substantially increase LTBI screening. However, CMS has not taken the administrative steps to include LTBI testing on their list of covered preventive services.^[18]^ This is a particular concern for Medicare beneficiaries because LTBI prevalence increases with age; accordingly, we found that Medicare beneficiaries had high rates of IGRA positivity (Table 1). Since Medicare is the largest third-party payer in the US, a CMS National Coverage Determination that adds LTBI testing to Medicare's list of covered preventive services would be a powerful lever to increase targeted screening.

While the majority of IGRA-positive persons had insurance, a sizable minority did not. Policy changes could move private sector providers to expand LTBI care to uninsured persons. State Medicaid plans may opt to cover treatment of LTBI-positive low-income persons who would otherwise be ineligible for Medicaid.^[19]^ Seven states currently elect this option; if more states included this option, it could help TB elimination efforts in the private sector. In addition, Medicaid 1115b waivers support use of Medicaid funds for special initiatives that could include uninsured persons, such as LTBI testing and treatment initiatives at some community health clinics.^[20]^ Our findings suggest such projects are useful, and their lessons learned will be important.

However, LTBI-related TB prevention efforts focused solely on community clinics would exclude many at-risk persons. Our results suggest that physician's offices/HMOs serve as the usual source of care for the largest proportion of non-US-born persons with LTBI. Local and state TB programs’ and national public health agencies’ engagement with private physicians and HMOs has the potential to increase targeted LTBI testing and treatment and greatly advance the nation's goal TB elimination goal.

Our analysis has limitations. Because the ACA was implemented after the 2011–2012 NHANES, post-ACA increases in insurance coverage and health care utilization were not captured,^[7]^ so our findings are likely underestimates. Additionally, the NHANES sample excludes noncivilian, institutionalized, and homeless persons, so these populations were not represented in our analysis. Hence, undocumented persons and refugees who recently arrived in the US may be underrepresented. We were also unable to examine IGRA positivity in children younger than 6 years, although young children with LTBI are highly susceptible to progression to TB. We did not have access to specific country of origin, so our data likely include non-US-born persons from countries with low TB incidence (e.g., Canada, Australia, western European countries). The relatively small sample of non-US-born persons resulted in unreliable estimates for certain categories of some variables. However, estimates of overall insurance coverage and having usual sources of care were robust and these results provide important, actionable insights.

## 5 Conclusion

Compared to the general US population, non-US-born persons face a higher TB risk and health care barriers. However, we found that health insurance and/or a usual source of health care are common among non-US-born individuals, including IGRA-positive individuals. These findings point to significant opportunities to advance TB prevention in the US by leveraging the reach and capacity of private health care.

## Acknowledgments

The authors gratefully acknowledge the support of the US Centers for Disease Control and Prevention's Division of Tuberculosis Elimination and its Tuberculosis Epidemiologic Studies Consortium (Atlanta, GA, USA) which provided valuable intellectual and other contributions. The authors extend a special thanks to Thomas R. Navin, Tracy Ayers, Ann M. Cronin, and Andrew N. Hill, who provided early feedback and guidance on the analyses, manuscript, and/or policy implications.

The findings and conclusions in this report are those of the authors and do not necessarily represent the official position of the United States Centers for Disease Control and Prevention (CDC). Mention of company names or products does not imply endorsement by the CDC.

## Author contributions

**Conceptualization:** Dolly Katz, Thaddeus L. Miller.

**Data curation:** Esther Annan.

**Formal analysis:** Esther Annan.

**Methodology:** Esther Annan, Erica L. Stockbridge, Dolly Katz, Thaddeus L. Miller.

**Project administration:** Erica L. Stockbridge, Dolly Katz, Thaddeus L. Miller.

**Supervision:** Erica L. Stockbridge, Dolly Katz, Eun-Young Mun, Thaddeus L. Miller.

**Validation:** Erica L. Stockbridge.

**Writing – original draft:** Esther Annan, Erica L. Stockbridge.

**Writing – review & editing:** Esther Annan, Erica L. Stockbridge, Dolly Katz, Eun-Young Mun, Thaddeus L. Miller.

## Supplementary Material

Supplemental Digital Content

## Supplementary Material

Supplemental Digital Content
