# Supplementary material for: A cross-sectional study of latent tuberculosis infection, insurance coverage, and usual sources of health care among non-US-born persons in the United States
Source: Medicine (Baltimore). 2021 Feb 19;100(7):e24838. doi: 10.1097/MD.0000000000024838 (PMC7899900; doi:10.1097/MD.0000000000024838)
Supplement: Supplemental Digital Content [file medi-100-e24838-s001.pdf]

ONLINE SUPPLEMENT #1 TO

Latent tuberculosis infection, insurance coverage, and usual sources of healthcare among foreign-born individuals in the United States

---

Methods Used to Generate Health Insurance and Usual Source of Health Care Variables

HEALTH INSURANCE

We used the following health insurance-related questions from the National Health and Nutrition Examination Survey (NHANES) to create a single primary health insurance variable. The new variable categorized coverage as Medicare, Medicaid/CHIP (but not Medicare), private insurance (but neither Medicare nor Medicaid/CHIP), other or unspecified insurance, or no insurance.

Question Text for HIQ.011:

The next questions are about health insurance.
Include health insurance obtained through employment or purchased directly as well as government programs like Medicare and Medicaid that provide medical care or help pay medical bills.
{Are you/Is SP} covered by health insurance or some other kind of health care plan?

Response Options	Numeric Code	Logic for Re-Coded Insurance Variable
Yes	1	Categorization depends on the answer to question HIQ.031. See the following page for details.
No	2	Categorized as “No insurance”
Refused	7	N/A: No non-US-born persons with interferon gamma release assay results refused to answer.
Don’t Know	9	N/A: No non-US-born persons with interferon gamma release assay results provided this response.

Question HIQ.031 was only displayed for persons answering “Yes” (Code 1) for HIQ.011]

Question Text for HIQ.031:

What kind of health insurance or health care coverage {do you/does SP} have? Include those that pay for only one type of service (nursing home care, accidents, or dental care). Exclude private plans that only provide extra cash while hospitalized. If {you have/s/he has} more than one kind of health insurance, tell me all plans that {you have/s/he has}. [ALL RESPONSES THAT APPLIED WERE CODED BY NHANES, UNLESS ANSWER 40 WAS SELECTED. NHANES ONLY ALLOWED ONE ANSWER WHEN 40 (NO COVERAGE OF ANY TYPE) WAS SELECTED.]

Response Options	Numeric Code	Logic for Re-Coded Insurance Variable
Private health insurance	14	Only considered if none of "Medicare," "Medicaid," or "SCHIP" were selected. If none of those options applied, categorized as "Private insurance"
Medicare	15	Categorized as "Medicare"
Medi-Gap	16	Not considered in logic; this is a Medicare supplement plan, so all persons selecting this option also selected "Medicare."
Medicaid	17	If "Medicare" not selected, categorized as "Medicaid/CHIP"
SCHIP (CHIP/Children's Health Insurance Program)	18	If "Medicare" not selected, categorized as "Medicaid/CHIP"
Military health services (TRICARE, VA, CHAMP-VA)	19	Only considered if none of "Private health insurance," "Medicare," "Medicaid," or "SCHIP" were selected. If none of those options applied, categorized as "Other or unspecified"
Indian health service	20	Only considered if none of "Private health insurance," "Medicare," "Medicaid," or "SCHIP" were selected. If none of those options applied, categorized as "Other or unspecified"
State-sponsored health plan	21	Only considered if none of "Private health insurance," "Medicare," "Medicaid," or "SCHIP" were selected. If none of those options applied, categorized as "Other or unspecified"
Other government program	22	Only considered if none of "Private health insurance," "Medicare," "Medicaid," or "SCHIP" were selected. If none of those options applied, categorized as "Other or unspecified"
Single service plan (e.g., dental, vision, prescriptions)	23	If this was the only option selected, categorized as "No insurance" (n=1). A single service plan would not cover the spectrum of services needed for latent tuberculosis infection testing and treatment.
No coverage of any type	40	Categorized as "Uninsured"

Response Options	Numeric Code	Logic for Re-Coded Insurance Variable
Refused	77	N/A: No non-US-born persons with interferon gamma release assay results refused to answer.
Don’t know	99	If no other options selected, categorized as “Other or unspecified”

Usual Source of Health Care

We used the following healthcare-related questions from the National Health and Nutrition Examination Survey (NHANES) to create a single usual source of health care (USHC) variable. The new variable categorized each person’s USCH as Clinic/Health Center, Dr. Office/HMO, Other/Not Specified, or No USHC.

Question Text for HUQ.030:

Is there a place that {you/SP} usually {go/goes} when {you are/he/she is} sick or {you/s/he} need{s} advice about {your/his/her} health?

Response Options	Numeric Code	Logic for Re-Coded USCH Variable
Yes	1	Categorization depends on the answer to question HUG.040. See the following page for details.
There is no place	2	Categorized as “No USHC”
There is more than one place	3	Categorization depends on answer to question HUG.040. See the following page for details.
Refused	7	N/A: No non-US-born persons with interferon gamma release assay results refused to answer.
Don’t know	9	N/A: No non-US-born persons with interferon gamma release assay results provided this response.

[Question HUQ.040 was only displayed for persons answering “Yes” (Code 1) or “There is more than one place” (Code 3) for HIQ.030]

Question Text for HUQ.040:

What kind of place {do you/does SP} go to most often: is it a clinic, doctor's office, emergency room, or some other place?

Response Options	Numeric Code	Logic for Re-Coded UCHC Variable
Clinic or health center	1	Categorized as “Clinic/Health Center”
Doctor’s office or HMO	2	Categorized as “Dr. Office/HMO”
Hospital emergency room	3	Categorized as “No USHC”
Hospital outpatient department	4	Categorized as “Other/Not Specified”
Some other place	5	Categorized as “Other/Not Specified”
Refused	7	N/A: No non-US-born persons with interferon gamma release assay results refused to answer.
Don’t know	9	N/A: No non-US-born persons with interferon gamma release assay results provided this response.
