# Supplementary material for: A cross-sectional study of latent tuberculosis infection, insurance coverage, and usual sources of health care among non-US-born persons in the United States
Source: Medicine (Baltimore). 2021 Feb 19;100(7):e24838. doi: 10.1097/MD.0000000000024838 (PMC7899900; doi:10.1097/MD.0000000000024838)
Supplement: Supplemental Digital Content [file medi-100-e24838-s002.pdf]

ONLINE SUPPLEMENT #2 TO

Latent tuberculosis infection, insurance coverage, and usual sources of healthcare among foreign-born individuals in the United States

---

<u>Methods Related to Complex Survey Design</u>

The National Health and Nutrition Examination Survey (NHANES) data were collected using a complex survey design that involved oversampling, stratification, and clustering. In accordance with the NHANES survey design, all data analyses were performed with Stata version SE 15.1 (StataCorp; College Station, TX, USA) using the 'SVY' functionality. Specifically, the following 'SVYSET' command preceded all data analyses:

```
svyset [w=wtmec2yr], psu(sdmvpsu) strata(sdmvstra)
```

This logic incorporated the following:

- It specified that the NHANES 2011-2012 mobile examination center sample 2-year weights (WTMEC2YR) be used in analyses. These weights adjusted for non-response to the physical examination and household interview, as well as the unequal probability of selection into the sample.
- It specified NHANES' 2011-2012 pseudo-primary sampling unit (SDMVPSU) and pseudo-stratum variable (SDMVSTRA) in order to account for the survey design's clustering and stratification.

In addition, our analyses were focused on the subpopulation of survey respondents who were non-US-born and who had interferon gamma release assay (IGRA) results. This subpopulation did not correspond to the NHANES design strata. Consequently, we used Stata's 'SUBPOP' option with the 'SVY' prefix commands. This option ensured that point estimates were based only on non-US-born persons with IGRA results, but the standard errors were calculated using all data as specified by the survey design variables described above. Without the 'SUBPOP' option, standard errors would have been incorrectly estimated.

In accordance with the National Health and Nutrition Examination Survey analytic guidelines (see reference #7), we deemed estimates for which the relative standard error (RSE) exceeded 30% to be unreliable. RSE was defined as the ratio of the standard error to point estimate.
